# Bringing the Heavy Chain to Light: Creating a Symmetric, Bivalent IgG-Like Bispecific

**DOI:** 10.3390/antib9040062

**Published:** 2020-11-06

**Authors:** Anusuya Ramasubramanian, Rachel Tennyson, Maureen Magnay, Sagar Kathuria, Tara Travaline, Annu Jain, Dana M. Lord, Megan Salemi, Caitlin Sullivan, Tristan Magnay, Jiali Hu, Eva Bric-Furlong, Pierrick Rival, Yanfeng Zhou, Dietmar Hoffmann, William Brondyk, Katarina Radošević, Partha S. Chowdhury

**Affiliations:** 1 Biologics Research, Sanofi R&D, 49 New York Avenue, Framingham, MA 01701, USA; rachel.tennyson@sanofi.com (R.T.); maureen.magnay@takeda.com (M.M.); sagar.kathuria@sanofi.com (S.K.); tara.travaline@gmail.com (T.T.); Annu.jain@cytiva.com (A.J.); dana.marie.lord@gmail.com (D.M.L.); Megan.Salemi@sanofi.com (M.S.); sullivcm@my.uri.edu (C.S.); tristan.magnay@sanofi.com (T.M.); jiali.hu@sanofi.com (J.H.); Eva.Bric-Furlong@sanofi.com (E.B.-F.); yanfeng.zhou@sanofi.com (Y.Z.); dietmar.hoffmann@sanofi.com (D.H.); whbrondyk@gmail.com (W.B.); 2 Biologics Research, Sanofi R&D, 13 Quai Jules Guesde, 94403 Vitry-sur-Seine, France; pierrick.rival@sanofi.com (P.R.); katarina.radosevic@lonza.com (K.R.)

**Keywords:** 2xVH, IgG-like bispecifics, bivalent bispecific molecule, symmetrical bispecific, concomittant binding, soluble VH

## Abstract

Bispecific molecules are biologically significant, yet their complex structures pose important manufacturing and pharmacokinetic challenges. Nevertheless, owing to similarities with monoclonal antibodies (mAbs), IgG-like bispecifics conceptually align well with conventional expression and manufacturing platforms and often exhibit potentially favorable drug metabolism and pharmacokinetic (DMPK) properties. However, IgG-like bispecifics do not possess target bivalency and current designs often require tedious engineering and purification to ensure appropriate chain pairing. Here, we present a near-native IgG antibody format, the 2xVH, which can create bivalency for each target or epitope and requires no engineering for cognate chain pairing. In this modality, two different variable heavy (VH) domains with distinct binding specificities are grafted onto the first constant heavy (CH1) and constant light (CL) domains, conferring the molecule with dual specificity. To determine the versatility of this format, we characterized the expression, binding, and stability of several previously identified soluble human VH domains. By grafting these domains onto an IgG scaffold, we generated several prototype 2xVH IgG and Fab molecules that display similar properties to mAbs. These molecules avoided the post-expression purification necessary for engineered bispecifics while maintaining a capacity for simultaneous dual binding. Hence, the 2xVH format represents a bivalent, bispecific design that addresses limitations of manufacturing IgG-like bispecifics while promoting biologically-relevant dual target engagement.

## 1. Introduction

The importance of antibodies as a therapeutic modality is now widely accepted as evidenced by the rapid rise of this drug class. Therapeutic monoclonal antibodies (mAbs), for instance, have maintained a dominant share of the drug market since 2017 and are expected to occupy nearly 20% of the drug market by 2022 [1,2]. Worldwide, over 570 mAbs have been studied in clinical trials and 97 have been approved in the US and Europe for a variety of indications [3,4]. Despite the commercial and clinical success of these molecules, the inherent monospecificity of mAbs is increasingly viewed as a bottleneck for their therapeutic potential. In particular, many diseases, including autoimmune disorders [5] and cancers [6,7], often have multiple immune modulators that cannot be effectively targeted by combinations of monospecific molecules and would therefore benefit from new mechanisms of actions associated with bi- and tri- specific molecules [8,9]. As such, the generation of bispecific antibodies, which take inspiration from natural monoclonal antibodies but are engineered for increased functionality, hold enormous clinical potential and address some of the limitations of therapeutic mAbs [9].

**Figure 1:**
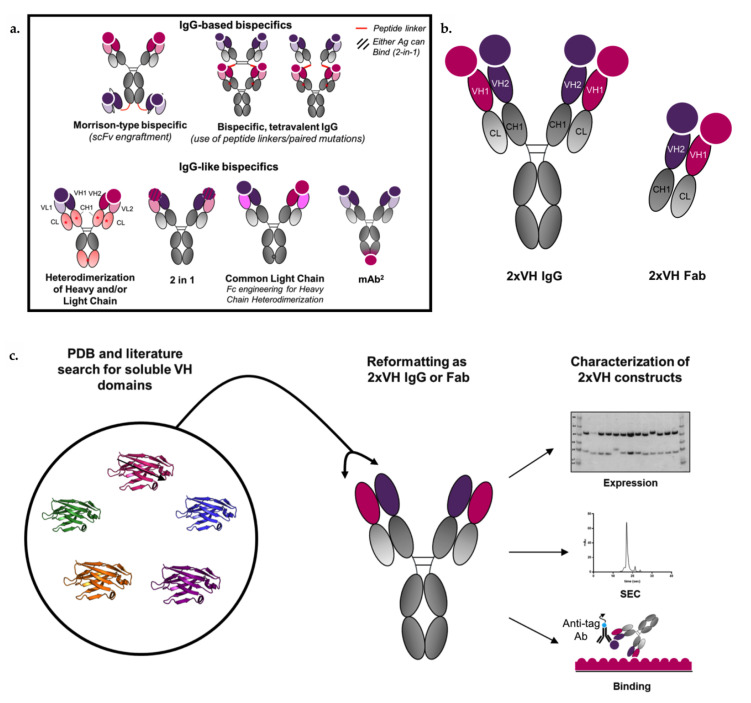
Examples of current bispecific IgG-based formats and overview of 2xVH IgG and Fab format. (**a**) Schematic depicting select examples of technologies used to generate IgG-based and IgG-like bispecifics including peptide linkers to generate tetravalent, bispecific IgGs, engraftment of scFvs on an IgG scaffold to generate Morrison-type bispecifics, heavy chain or light chain heterodimerization techniques, the 2-in-1 design, common light chain, and the utilization of Fcab via monoclonal antibodies (mAb^2^). Red asterisks refer to the presence of mutations while red lines refer to peptide linkers. The capacity of each bispecific molecule to engage the two target antigens, depicted as circles, is shown. Hashed circles for the 2-in-1 format indicate that either antigen can engage with the antibody paratope. (**b**) Representation of proposed 2xVH IgG and Fab structure and antigen binding. (**c**) Workflow for the generation and characterization of 2xVH constructs derived from publicly available VH domains.

Currently, over one hundred bispecific formats have been described in the literature [10], each with its strengths and weaknesses. These different formats can be classified as either fragment-based bispecifics or IgG-based bispecifics [11]. However, due to the short half-life of fragment-based bispecifics, there has been an increased focus on designing IgG-based bispecifics [12,13]. This latter class of bispecifics, examples of which are shown in Figure 1a, benefit from the Fc and an associated increased half-life while also leveraging existing manufacturing and formulation infrastructure of IgGs [14,15,16,17,18]. Among these IgG-based bispecifics, there are some that use IgGs as scaffolds and graft either scFvs or Fabs to these scaffolds to create symmetric molecules [19,20] while others are IgG-like and create different specificities in the two Fab arms. 

These asymmetrical IgG-like bispecifics possess potentially favorable drug metabolism and pharmacokinetic (DMPK) properties, owing to their similarities with traditional IgGs [17,18]. However, while these formats serve important niche applications, they suffer two important caveats: monovalent binding to each antigen and chain mispairing [11]. Since these IgG-like bispecifics bind each of their two targets in a monovalent fashion, they lose the target avidity of the monoclonal antibodies from which they are derived. Exceptions to this include the design proposed by Golay et al., which creates a tetravalent IgG-like bispecific using a CH1-CL heterodimerization strategy [19], or the Morrison type bispecifics that rely on grafting scFvs onto an IgG scaffold [21,22]. However, although these designs have been validated, they do not fully replicate the IgG structure. While there have been bispecific formats with a true IgG-like structure, such as the two-in-one antibody design [23] and the mAb^2^ design [24], that aim to create bivalency for each specificity, it is yet to be demonstrated whether bivalent, bispecific interactions can be mediated by either format in a concomitant manner. As an additional consideration, IgG-like bispecific antibodies require extensive engineering to ensure cognate chain pairing between the heavy and light chains [11]. Even with techniques like “knob-into-holes” [25], strand-exchange engineered domains [26] and Fab interface engineering [27,28,29], these molecules continue to face important manufacturing hurdles relating to low yields or the removal of chain mispaired byproducts. 

Here, we explore the possibility of generating a new bispecific design that aims to address the manufacturing and valency limitations of IgG-like bispecific molecules by leveraging the smallest immunoglobulin-based recognition unit, the VH domain. It has long been known that antigen-specific single human VH domains can exist in the absence of their cognate variable light domains (VLs) [30,31]. While stability of conventional IgGs depend on the VH/VL interaction, target-specific yet soluble and stable human or camelized human VH domains have been identified through both heavy-chain antibody (HcAb) transgenic mouse models [32,33,34] as well as phage display-based selections using semi-synthetic human VH libraries [35,36]. Recently, Shi et al. proposed a novel antibody format where two such soluble VH domains are used to replace the VH and VL of a traditional IgG through engraftment onto the CH_1_ and CL domains of each Fab arm (Figure 1b) [37]. This proof of concept study used two anti-β-klotho VH domains to create biparatopic IgG-like molecules that show synergistic agonism unobserved in the individual soluble VH domains. Additionally, since these molecules were symmetrically constructed using autonomous VH domains, no special cognate chain pairing was required. However, the format’s ability to tolerate individual VH domains targeting various antigens, a feature that would increase its broad, target-agnostic applicability, was unexplored. 

To assess the versatility of this format, referred to as 2xVH, we selected VH sequences with a range of sources and binding specificities from publicly available databases, including the protein databank (PDB). The criteria for selection included solubility following expression in cells, reasonable expression levels, a capacity for antigen binding and minimal surface hydrophobic patches as determined by BioVia Discovery Studio. Through this analysis, we selected thirteen previously identified VH domains. These domains were then characterized for expression and binding and further triaged before incorporation into the 2xVH IgG or Fab design. Once in the 2xVH format, the molecules were assessed for expression, purity and binding to their desired target antigens. Furthermore, we were able to validate dual binding in this format, including those targeting clinically relevant combinations, suggesting the modular nature of this format. Figure 1c schematizes this format modularity, particularly the capacity to integrate different VH domains identified from both in vitro and in vivo sources, as well as the initial steps for 2xVH molecule characterization. From our investigations, we believe the 2xVH format is a versatile bispecific, bivalent design that addresses some important limitations of generating IgG-like bispecifics [38] while showing promise as a dual- or multi-targeting therapeutic antibody format.

## 2. Materials and Methods

### 2.1. Selection of VH Domains from the Literature and the Protein Databank (PDB)

A review of both the protein databank (PDB) and the literature was performed to identify VH domains for production. The search included VH domains from in vitro display libraries [35,36] as well as in vivo sources including transgenic HcAb mice [32,34]. Relevant unique IDs from PDB were cross-referenced against the IMGT-3D structure-database for structural information [39], analyzed via BioVia Discovery Studio (Dassault Systèmes, Vélizy-Villacoublay, France) for hydrogen bonding, electrostatic interactions with its target antigen and surface hydrophobic patches. Structures were also annotated to identify chain and domain information. The VH domains from both PDB and literature searches were reviewed for their diversity of source (i.e., in vitro-derived or in vivo-derived) and the sequence availability to enable cloning. Domains were also ranked based on the commercial availability of target antigens. Additionally, available data from the literature regarding each domain’s expression, solubility and capacity to mediate antigen binding were also considered. Based on these parameters and our outlined structural analysis, a set of thirteen VH domains were cloned, expressed and characterized for binding and expression as described below. 

### 2.2. Expression and Purification of VH Domains

VH domains were cloned into a pHEN4 vector [40] using restriction enzymes NcoI and NotI (New England Biolabs, Ipswich, MA, USA). ExpiHEK293 cells were grown in 200 mL TB media (Sigma T-9179, Sigma-Aldrich, St. Louis, MO, USA) containing 0.8% glycerol, 2 mM MgCl_2_, 0.1% glucose and 100 μg/mL ampicillin at 37 °C. Protein expression was induced with 1 mM IPTG when cells reached an optical density of 0.6–0.9 (A_600_). Induction occurred at 28 °C, overnight, with shaking at 220 rpm. Cells were then pelleted and resuspended in 10 mL cold TES buffer. TES-suspended samples were placed on ice for 1 h. Simultaneously, TES buffer was diluted 4-fold in water and 15 mL of this diluted buffer was added to each sample and mixed via inversion. Samples were clarified via centrifugation at 5000× *g* for 30 min at 4 °C. Soluble lysate was allowed to bind 1 mL of Ni-NTA resin at 4 °C for 1 h while rotating. Resin was collected in disposable columns and washed with 5 column volumes (CV) of Buffer A (20 mM sodium phosphate, 500 mM NaCl, 5 mM imidazole pH 7.2). Proteins were then eluted with six CVs of Buffer B (20 mM sodium phosphate, 500 mM NaCl, 500 mM imidazole pH 7.2). Eluted pools were concentrated and buffer exchanged into Dulbecco’s Phosphate Buffered Saline (DPBS) using 3K MWCO ultrafiltration membrane tubes (MilliporeSigma, Burlington, MA, USA). Analyses of total protein concentration and protein purity were performed using the Bradford assay and SDS-PAGE, respectively. Final protein concentrations were reported based on NanoDrop microvolume spectrophotometry.

### 2.3. Expression and Purification of 2xVH Fabs and IgG Molecules

For the generation of recombinant 2xVH Fab and IgG plasmids, human-codon optimized gBlock^®^ DNA fragments were ordered from Integrated DNA Technologies (IDT) (Coralville, IA, USA). The gBlocks^®^ were then cloned into a pTT5 mammalian expression vector using In-Fusion^®^ Cloning technology (TaKaRa, Mountain View, CA, USA) to generate recombinant plasmids for heterologous expression in mammalian cells [41].

The fully automated mammalian cell secretory overexpression system, Protein Expression and Purification Platform (PEPP; GNF, San Diego, CA, USA), was used to express and to purify the bispecific antibody molecules. In brief, 35 mL of cultures seeded with 3.2 × 10^6^ Expi293F cells (Thermo Fisher Scientific A14527, Waltham, MA, USA) were transfected with 34 μg of plasmid DNA using the Expifectamine 293 transfection reagent per the manufacturer’s protocol (Life Technologies Corporation, Carlsbad, CA, USA). Cells were incubated at 37°C in an 8% CO_2_ gas environment for 5 days. Culture supernatants were harvested and applied to MabSelect SuRe protein A resin (GE Healthcare 17-5438-02, Chicago, IL, USA) for gravity flow purification. Proteins were desalted using Nap-10 sephadex (GE Healthcare 17-0854-02) and eluted with 1X DPBS (Thermo Fisher Scientific 14190136), resulting in a final storage solution of phosphate buffered saline, pH 7.4 for each protein sample.

### 2.4. SPR Analysis

All surface plasmon resonance (SPR) analyses were done on a Biacore T100 using HBS-EP+ (GE Healthcare Life Sciences BR100669, Marlborough, MA, USA) running buffer. For dual binding experiments, 2xVH IgGs were diluted to 5 µg/mL in running buffer and captured on a Protein A Series S Sensor chip (GE Healthcare Life Sciences 29127555, Marlborough, MA, USA). The antigens were diluted to 100 nM in running buffer and each was sequentially flowed over the captured 2xVH IgG. For 2xVH Fabs, goat anti-human F(ab’)2 (Jackson ImmunoResearch 109-005-006, West Grove, PA, USA) was amine coupled to a CM5 Series S sensor chip (GE Healthcare Life Sciences 29104988, Marlborough, MA, USA). The 2xVH Fabs were diluted to 20 µg/mL in running buffer and captured to the anti-F(ab’)2 surface. The antigens were diluted to 100 nM in running buffer and each was sequentially flowed over the captured 2xVH IgG or Fab. All data were double referenced, and the curves were evaluated for binding responses above baseline.

For kinetics experiments, 2xVH IgGs were diluted to 5 µg/mL, 2xVH Fabs were diluted to 20 µg/mL and VH domains were diluted to 10 µg/mL in running buffer. The 2xVH IgGs were captured on a Protein A Series S sensor chip, 2xVH Fabs were captured on the CM5 Series S sensor chip immobilized with goat anti-human F(ab’)2 and VH domains were captured on a CM5 Series S sensor chip immobilized with anti-his antibody (GE Healthcare Life Sciences 28995056, Marlborough, MA, USA). Antigens were diluted to 100 nM in running buffer and then serially diluted 2-fold for a total of six concentrations and a baseline, 0 nM control sample. Antigens were flowed over captured VHs from low to high concentration with a regeneration of 10 mM glycine-HCl pH 1.5 (GE Healthcare Life Sciences BR100354, Marlborough, MA, USA) in between each cycle. Resulting sensorgrams were double referenced and fit to a 1:1 binding model.

### 2.5. Analytical SEC

All size exclusion chromatography (SEC) analyses were done on an Agilent 1260 HPLC with a TSKgel G3000SWxl analytical column (Tosoh 0854, Tokyo, Japan) using phosphate buffered saline pH 6.8 as the mobile phase. For each sample, 20 µg of protein was injected in singlet over the column at 0.5 mL/min for 30 min and an absorbance at 280 nm (A280) was measured. Buffer blanks were included after each sample. Resulting chromatograms were blank subtracted and manually integrated to determine percent purity.

### 2.6. NanoDSF

Nano-format of Differential Scanning Fluorimetric (nanoDSF) was performed for purified VH domains using Prometheus NT (NanoTemper technologies PR001, Cambridge, MA, USA). Intrinsic tyrosine and tryptophan fluorescence were measured during thermal denaturation from room temperature to 95 °C at 1 °C/min ramp and 30% intensity.

### 2.7. ELISA

For dual-binding ELISAs, the surface immobilized antigen was coated overnight at 4 °C on an Immulon 4 HBX plate (Thermo Fisher Scientific 3855, Waltham, MA, USA) at 5 μg/mL. The plate was then washed three times with Phosphate Buffered Saline (PBS) solution using an automatic 96-well microplate washer (Titertek Berthold, Pforzheim, Germany) and blocked with 50 μL of 3% milk-PBS for 30 min. The blocked plates were subsequently washed twice with PBS supplemented with 0.1% Tween and once with PBS before varying concentrations of 2xVH IgG or Fab, diluted in 3% milk-PBS, were added to each well of the microtiter plate. The plates were incubated for one hour and subsequently washed twice with PBS supplemented with 0.1% Tween and once with PBS. The second tagged antigen, diluted to 5 μg/mL in 3% milk-PBS, was then added to the plate and incubated for 1 h before the plates were washed as previously described. Eu-labelled secondary antibody (Perkin Elmer, Waltham, MA, USA) targeting the tag (anti-Biotin, anti-His or anti-Fc) was then added at 0.2 μg/mL in DELFIA assay buffer (PerkinElmer, Waltham, MA, USA) and microtiter plates with secondary antibody were allowed to incubate in the dark for one hour. Plates were subsequently washed with DELFIA wash buffer and developed using the DELFIA enhancement solution according to the manufacturer’s instructions. After 30 min of incubation, the fluorescence was measured via a microtiter plate reader at 615 nm.

### 2.8. 2xVH Fab Crystallization

Recombinant 2xVH anti-Lysozyme/Her2 Fab was expressed in Expi293F cells and purified by MabSelect SuRe protein A resin (GE Healthcare 17-5438-02, Chicago, IL, USA) followed by size exclusion chromatography (Superdex 200 10/300 GL-GE Healthcare; buffer: 20 mM HEPES pH 7.0, 50 mM NaCl). The protein was concentrated to 10 mg/mL and crystallized at room temperature in 1 M lithium chloride, 0.1 M citric acid pH 4.0. These crystals were cryoprotected in 20% ethylene glycol and mother liquor. X-ray diffraction data were collected at the Candian Macromolecular Crystallography Facility (CMCF) at the Canadian Light Source (CLS) using a Pilatus 3 6 M detector (Dectris, Philadelphia, PA, USA). The data were processed using XDS [42] and Aimless [43]. Molecular replacement was performed using Phaser and the data were refined using phenix.refine and the crystallographic object-oriented toolkit (COOT) [44,45,46]. Data collection and refinement statistics are listed (Table S1). Software used in this project was accessed through the SBGrid consortium [47]. Atomic coordinates have been deposited in the Protein Data Bank under accession code 7JKB.

## 3. Results

### 3.1. Identification of In Vitro and In Vivo-Derived Candidate VH Domains

**Figure 2:**
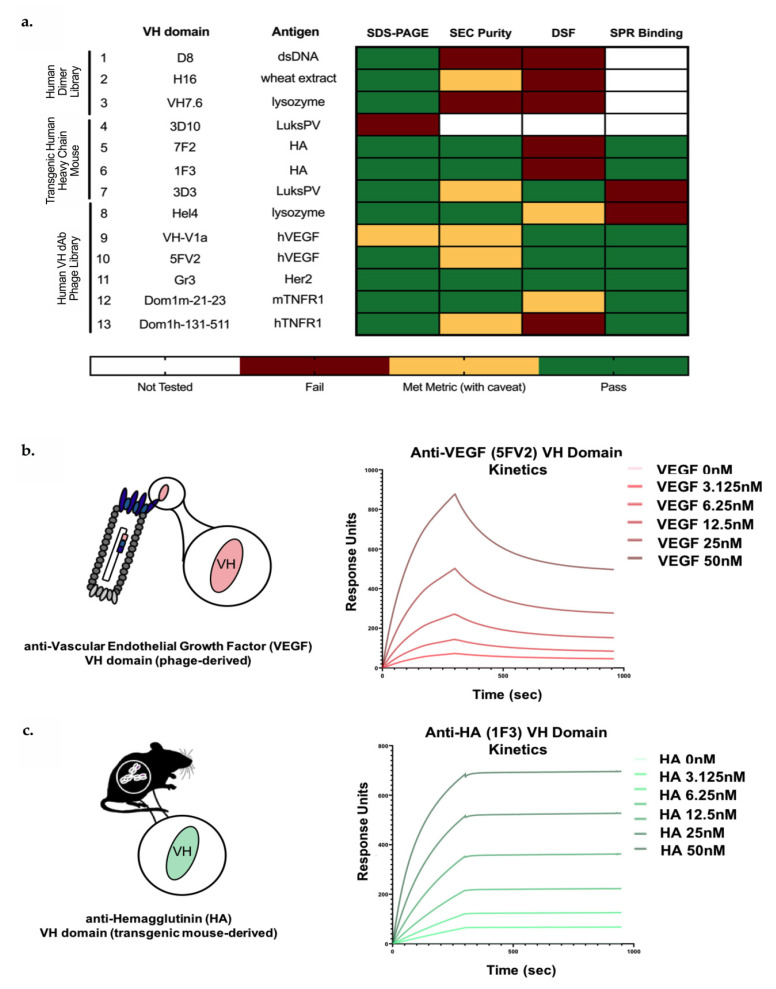
Shortlist of VH domains identified from literature and protein databank search. (**a**) A heatmap summarizing the thirteen VH domains from the literature chosen for further biophysical characterization. VH domains were characterized and triaged based on their expression, purity, melting temperature (*Tm*), and binding. Expression was assessed by recombinant protein production and visualization of soluble protein on an SDS-PAGE gel. Soluble protein was characterized as having passable expression (green), if the protein expressed at the correct molecular weight. VH domains that met this metric with caveat, shown in yellow, expressed but at an unexpected molecular weight, whereas those that failed (red) showed no protein expression. Likewise, for size exclusion chromatography (SEC), VH domains with a monomeric purity of 95% or higher are marked as green, while those that were 90–94.9% monomeric or ran at unexpected weight were marked in yellow. Any VH domain that was <90% monomer was flagged (red). Melting temperatures, *T_m_*, were determined by nano-DSF and used as a measure of thermal stability. VH domains with a *T_m_* above 60 °C were the most thermostable (green). Any VH domains with a *T_m_* between 57.5–59.5 °C were considered moderately thermostable (yellow), while domains displaying *T_m_*s below 57.5 °C had poor thermostability and are marked in red. Binding was analyzed by SPR for expressible VH domains and either marked in green for observable binding or in red for no observed binding. (**b**) Binding kinetics for phage-derived VH domain, 5FV2, binding to its target antigen, human vascular endothelial growth factor (VEGF). (**c**) Binding kinetics for transgenic mouse-derived 1F3 binding to its target antigen, hemagglutinin (HA). Both VH domains bind their target with affinities of 3 nM and 0.7 nM respectively.

To understand the capacity of the modular 2xVH bispecific IgG to tolerate diversely-sourced VH domains, we searched the protein databank (PDB) and the literature for soluble VH domains discovered through both in vitro and in vivo antibody discovery methods. Despite the generally poor biophysical properties of autonomous VH domains [32,34,35,36], our in silico search yielded thirteen soluble, human VH domains targeting commercially available antigens as seen in Figure 2a. Interestingly, eleven of the thirteen VH domains were members of the VH3 family—a master framework known to be more soluble and stable than any other human VH group in the absence of a cognate VL [48]. Despite this similarity, VH domains were derived from three distinct sources, namely phage-display libraries, transgenic mice or VH dimer (VHD) libraries, and some of the unique features of each class of domains are described below.

In vitro sources, like phage display, can be used to identify non-aggregating VH domains against desired target antigens through panning synthetic or semi-synthetic libraries based on master frameworks like the V3-23 germline [49]. Analysis of published soluble VH (sVH) crystal structures in BioVia Discovery Studio suggested that incorporation of hydrophilic residues at the VL interface reduces hydrophobicity and can further improve solubility and any aggregation-associated issues that persist with these domains. In some cases, mutations of just the complementarity-determining regions or CDRs can be sufficient to induce a small conformational change that shields or alters a hydrophobic patch at the VH interface and thereby solubilizes the VH domain [50]. The solubilizing effect of mutations in the CDR was seen for one of our candidate VH domains, HEL4—a molecule that contains negatively charged residues in its CDR1 that causes a framework tryptophan to rotate inward. The rotation of this hydrophobic tryptophan side chain was proposed to have improved both the solubility and stability of HEL4 [51].

Although such solubilizing mutations can be rationally introduced to the CDRs of VH domains [52], phage display libraries also afford the opportunity to leverage stress-selections by heat [35], protease or acid treatment [53] to identify and produce stable VH domains. Previously, it was shown that three rounds of stress-selections on an error-prone PCR library derived from one of our candidate VH domains, Dom1h-131-511, could produce protease-optimized clones [54]. One such clone, DOM1h-131-203, showed a two-fold improvement in binding affinity for hTNFR1 and an increased melting temperature, a hallmark of improved thermostability among IgGs and VH domains [50,52]. As a consequence of this enormous flexibility afforded by phage display in tailoring library-based selections, numerous soluble VH (sVH) domains have been reported and six different phage-derived sVH domains were shortlisted for introduction in the 2xVH format—5FV2 targeting human VEGF [55], Dom1h-131-511 targeting human TNFR1 [54], Dom1m-21-23 targeting mouse TNFR1 [56], Gr3 targeting Her2 [57], Hel4 targeting lysozyme [35] and VH-V1a targeting human VEGF [58]. The criteria used for the selection of these VH domains as well as our in vivo-derived VH domains are outlined in the Materials and Methods.

In addition to in vitro discovery techniques, in vivo sources, like transgenic mice containing hybrid llama/human Ig heavy chains, can also produce HcAb upon immunization. Work by Drabek et al. showed that the VH portion of these HcAbs can be isolated and expressed as soluble proteins [59]. Upon sequence analysis, Drabek and colleagues found an increase in net hydrophobicity and the presence of charged residues in select CDR and framework regions, a feature that facilitates the solubility of these fully human VH domains. Consequently, four different transgenic mouse-derived VH domains bearing similar charged residues—1F3 and 7F2 targeting hemagglutinin [59], 3D3 and 3D10 targeting LuksPV [60]—were identified in silico and selected by us for further characterization and incorporation into the 2xVH format.

Aside from in vitro and in vivo discovery of native sVH domains, we were also interested in considering in vitro-derived VH dimers. It has been hypothesized that VH domains have the capacity to dimerize owing to the structural similarity between VH and VL and the capacity of the latter to form homodimers [61]. These VHDs create a VH:VH interface that is an appealing feature for the 2xVH format and similar in structure to a native VH:VL interface. Given this feature of VHDs, we decided to explore the capacity of VHD-derived VH domains to generate similar stabilizing interactions in the 2xVH context through three such domains: D8 targeting dsDNA [62], H16 targeting wheat extract [61] and VH7.6 targeting lysozyme [63]. Consequently, thirteen VH domains were incorporated into the 2xVH format, six of which were derived from the phage platform, four from transgenic mice and three from phage-derived VH dimer libraries.

### 3.2. Biophysical Characterization of sVH Candidates Shows Source-Dependent Differences in Expression, Purity and Thermal Stability

We hypothesized that three key biophysical parameters—expression, purity and thermal stability—of soluble VH domains would have an important influence on the production of any derived 2xVH bispecific molecule. Therefore, each of the thirteen domain antibodies was expressed in a small-scale bacterial culture system, purified from the periplasm, and characterized via SDS-PAGE. Five of the six VH domains identified through phage-display uniformly expressed as soluble proteins by SDS-PAGE and all but one, VH-V1a being the exception, showed bands at their expected molecular weight. Of the four VH domains derived from transgenic mice, all but one, 3D10, could be expressed as a soluble protein. Similarly, all three VHD-derived VH domains showed uniform expression as soluble protein by SDS-PAGE. At this point, any VH domain that could not be easily produced in bacteria was triaged out, leaving twelve VH domains that were further characterized.

Measures of protein heterogeneity or purity of the monomeric fraction are essential for both antibodies and antibody fragments since the presence of impure species can result in aggregation [64] and other undesirable properties. To better understand the biophysical characteristics of these twelve soluble VH domains, we moved forward with subsequent analysis of purity by analytical size exclusion chromatography (aSEC) as shown in the classification scheme in Figure 2a. VH-V1a, despite having an SEC purity of 95.5%, was an exception to this classification scheme. Analysis by aSEC suggested the monomer was larger than anticipated, a finding that was further supported by SDS-PAGE analysis (data not shown). While the majority of phage- and transgenic-mouse derived VH domains showed a single monomeric peak in SEC chromatograms and purities above 90%, only one VHD-derived sVH (H16) showed comparable purity by aSEC (91.5% monomeric). By contrast, two VHD-derived VH domains, D8 and VH7.6, demonstrated a broad peak by aSEC, indicative of possible aggregates or unwanted isoforms of the VH domain [64]. In fact, these poor SEC profiles are corroborated by previous work illustrating the hydrophobicity of VHD dimerization interface [31]—a feature which, unless engineered or masked by pairing, could result in aggregation of the monomeric VH. Consequently, these molecules were flagged for triaging and are marked in ‘red’ in the heatmap in Figure 2a.

As with measures of protein aggregation, protein stability is a key developability parameter that can provide insight into the capacity of individual VH domains to maintain structural integrity as soluble domains and in the context of a bispecific format. Here, protein stability was addressed through thermal unfolding experiments where individual VH domains were subjected to a linear temperature ramp and thermal stability, quantified by the melting temperature (*T_m_*), was monitored by nanoDSF. For the twelve identified VH domains the *T_m_*s ranged from 47 °C to 65.4 °C, with six molecules showing higher *T_m_*s above 58 °C. Unsurprisingly, VH domains identified from optimized phage display and transgenic mice showed higher *T_m_*s (ranging from 55.9 to 65.2 °C and 54 to 57.1 °C, respectively), further supporting the idea that stress-selections and interface engineering play an important role in increasing the stability of these molecules. This is illustrated by DOM1h-131-511, a native VH domain targeting hTNFR1 that we initially shortlisted for incorporation in our bispecific 2xVH format. However, as suggested by Enever et al., this molecule showed a low *T_m_* (<58 °C) and monomeric purity (<90%) by aSEC (data not shown) [54]. Consequently, we instead decided to move forward with the protease-optimized variant, DOM1h-131-203, reported to have a higher *T_m_* of 64.4 °C by differential scanning calorimetry (DSC). We also observed that VHD-derived VH domains had the lowest *T_m_*s at 47 and 53 °C, and these low melting temperatures, coupled with poor purity by SEC, eliminated these domains as candidates for the 2xVH format.

### 3.3. Candidate In Vitro- and In Vivo-Derived sVH Domains Show nM and Sub-nM Binding

Before generating bispecific molecules, we assessed if the individual sVH domains mediate strong, specific binding for their target antigen. To understand the kinetic parameters between each VH domain and corresponding target antigen, the nine shortlisted molecules were assessed by SPR and individually captured and oriented on the SPR chip surface before saturating concentrations of the cognate antigen were flowed over the chip. Two examples of VH kinetics, one for the phage-derived 5FV2 and another for the transgenic mouse-derived 1F3, are shown in Figure 2b,c. Fitting both these sensorgrams with a 1:1 binding model indicates high affinities of 3 nM for the 5FV2-hVEGF interaction and 0.7 nM for the 1F3–HA interaction. The high affinities can be attributed to 5FV2’s fast on-rate (7.3 × 10^5^ M^−1^s^−1^) and 1F3’s slow off-rate (5.5 × 10^−5^ s^−1^). Figure S1 illustrates the binding interactions of the remaining seven VH domains showing measurable equilibrium dissociation constants (*K_D_*) by SPR. The affinities of the different VHs for their cognate antigens ranged from 500 nM to 2.7 pM.

While these nine phage- and HcAb transgenic mouse-derived VH domains showed stable interactions with their target antigen, characterized by nM or sub-nM *K_D_*s, two domains, 3D3 targeting Luks PV and HEL4 targeting lysozyme, were unable to bind their targets. Though the absence of target-specific binding was in contrast to the reported literature [34,35], it is possible that both VH domains require oriented antigen presentation, as suggested by Friguet et al. [65], or multivalency [35] to initiate binding to their target. While we did not assess the capacity to rescue binding through alternate antigen or VH presentations, both 3D3 and Hel4 displayed favorable purity and thermostability as monovalent sVH (Figure 2a). This suggests that elements of the 3D3 and Hel4 framework, derived from the VH3-23 germline, may confer stability and potentially serve as a scaffold for further engineering. For this reason, both 3D3 and Hel4 were pursued alongside the other seven VH domains for reformatting and assessment in the 2xVH format. Since two VH domains targeting VEGF and two targeting HA had been shortlisted, the anti-VEGF and anti-HA VH domains with lower SEC purity and *T_m_*s, VH-V1a and 7F2, were triaged out. The seven, unique VH domains that ultimately progressed toward incorporation in the 2xVH scaffold were 1F3, 3D3, Hel4, 5FV2, Gr3, Dom1m-21-23 as well as the protease-optimized variant of Dom1h-131-511, Dom1h-131-203.

### 3.4. Generation and Characterization of 2xVH Molecules with High Yield and Purity

**Figure 3:**
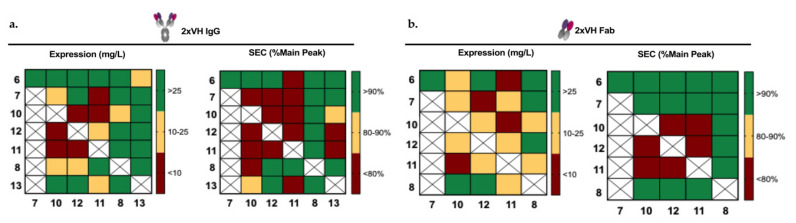
Characterization of 2xVH IgG and Fab expression and purity. (**a**) Heat map denoting the expression and purity of 2xVH IgG. VH_1_, corresponding to the heavy chain position on the native IgG, is listed on the vertical axis of each heatmap while VH_2_, corresponding to the light chain position, is listed on the horizontal axis of the heatmaps. The identity of the VH domain is delineated by a number scheme identical to the one outlined in Figure 2a. The expression, extrapolated into mg/L, is denoted in green for those IgGs expressing at greater than 25 mg/L, yellow for those showing moderate expression of 10–25 mg/L and red for any molecules with poor expression below 10 mg/L. SEC purity was calculated as a percentage of area under the main peak with 90% or higher monomeric purity marked in green, 80–90% monomeric purity marked in yellow and below 80% purity in red. Duplicate pairings (i.e., pairings with two identical VH domains) or combinations that were not produced are marked in white. (**b**) Heat map denoting expression and purity of corresponding 2xVH Fabs. Expression and SEC criteria were the same for both the 2xVH IgGs and Fabs.

We were interested in understanding how each of these domains would behave when paired with another VH in the 2xVH format. To explore the degree of modularity in the 2xVH design, each of the seven characterized VH domains were combinatorially paired with the remaining six VH domains to generate twenty-one two-domain combinations. By accounting for two possible placements for each VH domain within the 2xVH format, forty-two unique 2xVH molecules were designed. Due to constraints in cloning and expression capacity, 31 IgGs and 21 Fabs were generated as illustrated in Figure 3.

Codon-optimized Fab- and IgG-versions of these molecules were cloned into a mammalian expression vector, expressed in small-scale cultures and assessed for expression and purity as shown by the heatmaps in Figure 3a,b, respectively. It is important to note that although our constituent sVHs were expressed and screened in *E. coli*, a flexible system for the expression of small or simple proteins, we chose to express the final 2xVH constructs in mammalian Expi293F cells since the expression of complex molecules like full-length IgGs is often difficult, if not impossible, in bacterial systems [66,67,68]. Overall, mammalian cell-expressed 2xVH Fabs showed similar expression with consistently higher purity compared to the corresponding 2xVH IgGs as seen in Figure 3b. Regardless of whether the VH pairs were expressed as Fabs or IgGs, certain VH_1_–VH_2_ combinations showed high expression and purity while other combinations showed poor tolerance for the bispecific format. For example, IgGs and Fabs with transgenic mouse-derived 1F3 in the VH_1_ position or the phage-derived Hel4 and DOM1h-131-203 in the VH_2_ position showed yields greater than 25 mg/L and over 90% purity. By contrast, other phage-derived VH domains including 5FV2 and Gr3, while stable as soluble VH fragments, showed poor expression and purity as 2xVH except when paired with 1F3, Hel4 or DOM1h-131-203.

To further investigate the stabilizing effects of these three VH domains, a small panel of molecules incorporating these domains—an anti-HA/anti-hVEGF (i.e., 1F3/5FV2), both orientations of the anti-hTNFR1/anti-hVEGF (i.e., 5FV2/DOM1h-131-203) and an anti-lysozyme/anti-Her2 (i.e., 5FV2/DOM1h-131-203)—were assessed for purity, binding and structure. The learnings from representative 2xVH constructs are discussed in the following sections.

### 3.5. 2xVH IgG and Fab Can Co-Engage Two Antigens: A Case Study of Anti-HA/Anti-hVEGF

**Figure 4:**
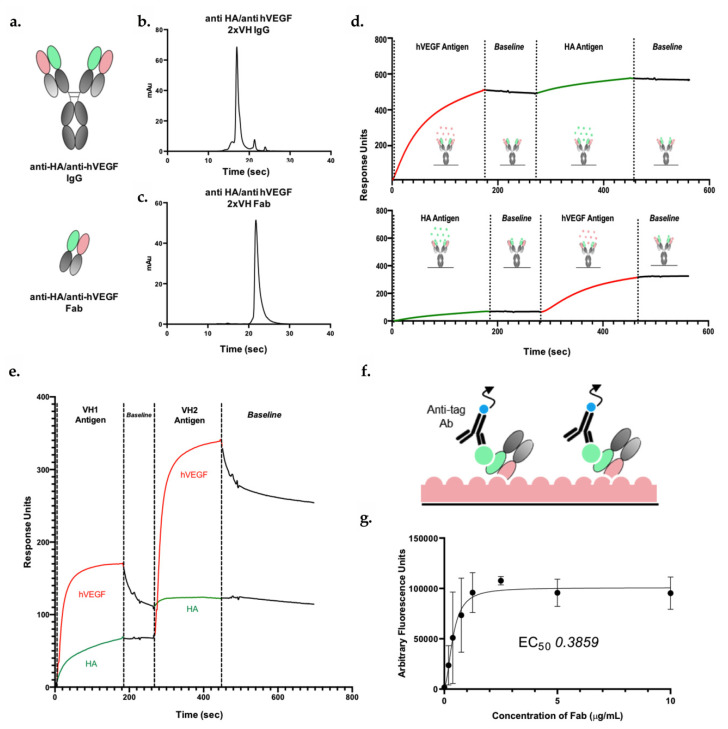
2xVH format allows for dual target engagement as IgG and Fab. (**a**) Schematic of anti-HA/anti-hVEGF 2xVH IgG and Fab. (**b**,**c**) SEC profile of anti-HA/anti-hVEGF IgG and Fab. (**d**,**e**) SPR analysis of anti-HA/anti-hVEGF IgG (**d**) and Fab (**e**) show binding for corresponding antigens regardless of the order of antigen injection. (**f**) Schematic of the time-resolved fluorescence ELISA for determining the capacity of the 2xVH Fab to engage simultaneously with two target antigens. In this sandwich ELISA, an untagged target antigen is coated on the surface before interacting with its 2xVH Fab at varying concentrations (0–10 g/mL). A second, tagged antigen is subsequently introduced and allowed to bind the complex. Secondary antibodies recognizing that tag are then introduced to probe the antibody–dual antigen complex. A fluorescence signal at 655 nm occurs only if the Fab can bind both antigens at the same time. By contrast, the absence of a streptavidin-based signal suggests that both antigens cannot simultaneously bind the Fab, either because the Fab is unable to bind the surface-bound antigen (HA) or because second biotinylated antigen (hVEGF) is unable to bind to the Fab-HA complex. (**g**) ELISA results show concentration dependent binding of anti-HA/anti-hVEGF Fab to its corresponding antigens, HA and VEGF.

To gain further understanding of the binding capacity of the 2xVH format and to ensure that VH domains originating from both in vivo and in vitro sources could integrate into a single 2xVH molecule, we generated prototype Fab and IgG molecules using a VH from transgenic mice, 1F3, and an optimized, phage-derived VH domain, 5FV2, as shown in Figure 4a. 1F3 was grafted onto CH1 of the heavy chain (VH_1_ position) and 5FV2 was grafted onto the CL domain of the light chain (VH_2_ position). Anti-HA/anti-hVEGF molecules expressed well as an IgG and showed a single peak by aSEC as seen in Figure 4b. While the Fab fragment expressed just shy of 25 mg/L, the molecule nevertheless eluted as a single peak (Figure 4c).

Next, to interrogate if these molecules could engage both their targets, HA and hVEGF, the 2xVH IgG and Fab molecules were assessed for binding by SPR. For this analysis, each 2xVH molecule was captured on an SPR sensor chip surface and sequentially exposed to each target antigen. We also assessed how dual binding differed if the molecule was first exposed to the 46 kDa hVEGF homodimer followed by the larger, 85 kDa HA as opposed to HA followed by hVEGF. While both the anti-HA/ anti-hVEGF 2xVH IgG and the 2xVH Fab showed the capacity to bind the two target antigens, the binding profiles of the IgG and Fab differed significantly as seen in Figure 4d,e. In Figure 4d, the 2xVH IgG showed stable engagement and limited dissociation from both antigens regardless of the order in which the two targets were flowed over the IgG sample. By contrast, as shown in Figure 4e, hVEGF shows a partial, rapid dissociation from the 2xVH Fab both in the presence of existing HA interactions and in isolation. Furthermore, Fab engagement with HA was four-fold more attenuated when hVEGF interactions were already present than when HA was the first antigen to bind the 2xVH Fab. This suggested that while both 2xVH formats were able to engage with target antigens, the 2xVH IgG, by virtue of its bivalency, was able to cross-link VEGF homodimers and display stable antigen engagement. By contrast, the 2xVH Fab—a monovalent format whose binding capacity is more severely impacted by steric effects than the bivalent IgG—had more limited, sterically-driven target coengagement. Incidentally, this corroborates with the original characterization of 5FV2 performed by Walker et al. where a bivalent construct, consisting of two 5FV2 VH domains joined by a Gly-Ser linker, was shown to be more effective at binding and capturing the VEGF homodimer than a single, monovalent 5FV2 [55].

To explore if the 2xVH anti-HA/anti-hVEGF Fab could, in fact, simultaneously coengage with both hVEGF and HA, a modified sandwich ELISA assay was employed as shown in Figure 4f. In this scheme, the 2xVH Fab, at varying concentrations, was allowed to bind surface-adsorbed, untagged HA. Biotinylated hVEGF was subsequently allowed to interact with the Fab–HA complex and the entire trimeric complex was probed by a streptavidin-conjugated secondary antibody through a time-resolved fluorescence assay. Unlike with SPR, a positive signal is only observed in the sandwich ELISA when both HA and hVEGF bind to a single Fab. Using this fluorescence-based readout, Figure 4g illustrates that 2xVH anti-HA/anti-hVEGF Fab was able to mediate a dose-dependent, simultaneous co-engagement with both HA and hVEGF. Consequently, this binding data unambiguously suggest that both target antigens can simultaneously engage with their VHs within the same Fab arm of a 2xVH molecule—the first such illustration of simultaneous, dual binding for this format.

### 3.6. VH Domain Placement Does Not Impact Binding to Target Antigen in 2xVH Format: A Case Study of Clinically Relevant Anti-hVEGF/Anti-hTNFR1

**Figure 5:**
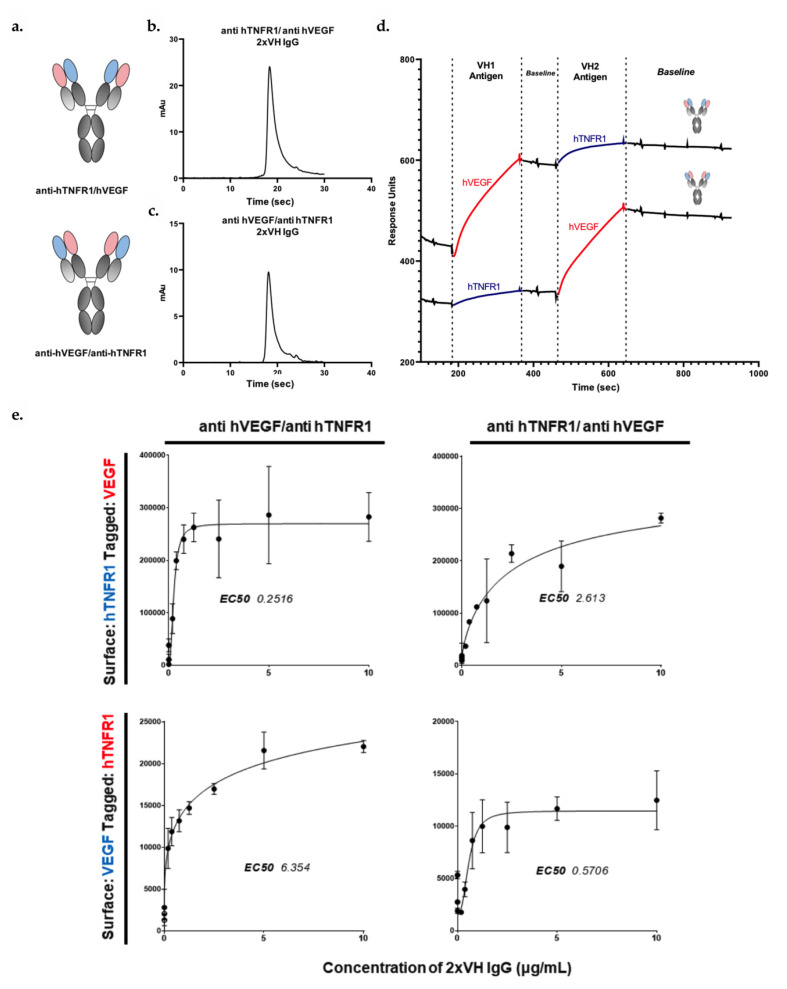
The 2xVH IgGs can undergo dual binding but domain placement and order of antigen binding can alter half-maximal effective concentrations or EC_50_s (**a**) Schematic of anti-hTNFR1/anti-hVEGF and anti-hVEGF/anti-hTNFR1 2xVH IgGs. (**b**,**c**) Traces of monomeric purity for Anti-hTNFR1/anti-hVEGF IgG and hVEGF/anti-hTNFR1 IgG as assessed by analytical SEC. (**d**) SPR traces showing the sequential binding to the VH_1_ and VH_2_ antigen for the two mirror-image molecules, anti-hTNFR1/anti-hVEGF and anti-hVEGF/anti-hTNFR1 2xVH IgGs. (**e**) Time-resolved fluorescence ELISA results showing concentration dependent binding of the two mirror-image molecules, anti-hTNFR1/anti-hVEGF and anti-hVEGF/anti-hTNFR1. The top panels show dual binding of the two molecules when hTNFR1 is immobilized on the surface and hVEGF is tagged. In the bottom panels, the molecules are introduced to a hVEGF-immobilized surface and probed with tagged hTNFR1. While both 2xVH IgG can bind target antigens and display dual binding, domain placement and order of antigen binding can alter the EC_50_ tenfold.

Having shown that prototype 2xVH molecules can engage in simultaneous, dual binding, we asked whether domain placement within the IgG could impact a molecule’s capacity to bind its two, target antigens. To this end, two 2xVH IgGs incorporating 5FV2 targeting hVEGF, a mediator of angiogenesis in both development and disease, and DOM1h-131-203 targeting hTNFR1, the canonical receptor for the inflammatory TNF cytokine family, were generated and characterized. Notably, blocking both hVEGF and hTNF signaling has been observed to have therapeutic benefits in a number of autoimmune disorders including rheumatoid arthritis [69,70]. In the first molecule targeting these two antigens, the 5FV2 domain was grafted onto the CH1 domain or the VH_1_ position while DOM1h-131-203 was grafted onto the CL, in the VH_2_ position. In the inverted anti-hTNFR1/anti-hVEGF, the orientations were reversed and DOM1h-131-203 occupied the VH_1_ position while the 5FV2 was grafted onto the VH_2_ position. The schematics for these two molecules are shown in Figure 5a. Although 2xVH IgGs expressed well and their SEC profiles look to be 90% pure, we observed some minimal peak tailing suggesting column interactions and the possible presence of unresolved low molecular weight contaminants (Figure 5b,c). To address these concerns, SEC traces for both molecules were fit with exponentially modified Gaussian (EMG) curves as shown in Figure S2. While these fits suggest the possible presence of low-molecular weight contaminants, more than 70% of both samples are pure, monomeric IgG.

The initial binding assessment for these molecules was done by both SPR and ELISA. As illustrated in Figure 5d, both 2xVH IgGs show a similar capacity to engage in stable, dual binding by SPR when presented first with the VH_1_ antigen followed by the VH_2_ antigen. However, as with the prototype anti-HA/anti-hVEGF molecules, SPR-based analysis provides limited insight into whether the 5FV2- and DOM1h-131-203-containing bispecific can bind two antigens simultaneously or if these binding events are spatially and temporally distinct. To address this question and understand if these molecules engage antigens simultaneously, we employed a similar sandwich ELISA strategy used to characterize dual binding for the 2xVH anti-HA/anti-hVEGF Fab. In short, each 2xVH molecule could interact with either surface-immobilized hTNFR1 or hVEGF. The surface immobilized antigen-2xVH complex was then allowed to interact with the second tagged-antigen and probed with a secondary antibody targeting this tag. Figure 5e illustrates how each 2xVH IgG anti-hTNFR1/anti-hVEGF and anti-hVEGF/anti-hTNFR1 mediates dual binding when the VH_1_ antigen is surface-immobilized and the VH_2_ antigen is tagged, as well as, when the VH_2_ antigen is surface-immobilized and the VH_1_ antigen is tagged. While both 2xVH IgGs showed significant, dose-dependent dual binding, the measured EC_50_s for these interactions varied depending on the presentation of the antigen. Namely, when the VH_2_ antigen was surface-immobilized and initiated dual binding to the 2xVH molecule, the measured EC_50_ for dual binding was ten-fold lower than when the VH_1_ antigen was surface immobilized and the 2xVH molecule sequentially bound via VH_1_ followed by VH_2_. This suggests that this 2xVH molecule participates in a stronger dual binding response when the VH_2_ antigen initiates dual binding. Conversely, when the VH_1_ domain interacts with its target antigen first, the 2xVH molecule may have a weaker, subsequent interaction with the VH_2_ antigen. While both 2xVH molecules mediated dual binding, the quality of this binding interaction can be influenced by sterics and vary based on both the domain placement and the order of antigen binding.

### 3.7. Anti-Her2/Anti-Lysozyme: VH/VH Interfaces Are Larger than Canonical VH/VL Interfaces

**Figure 6:**
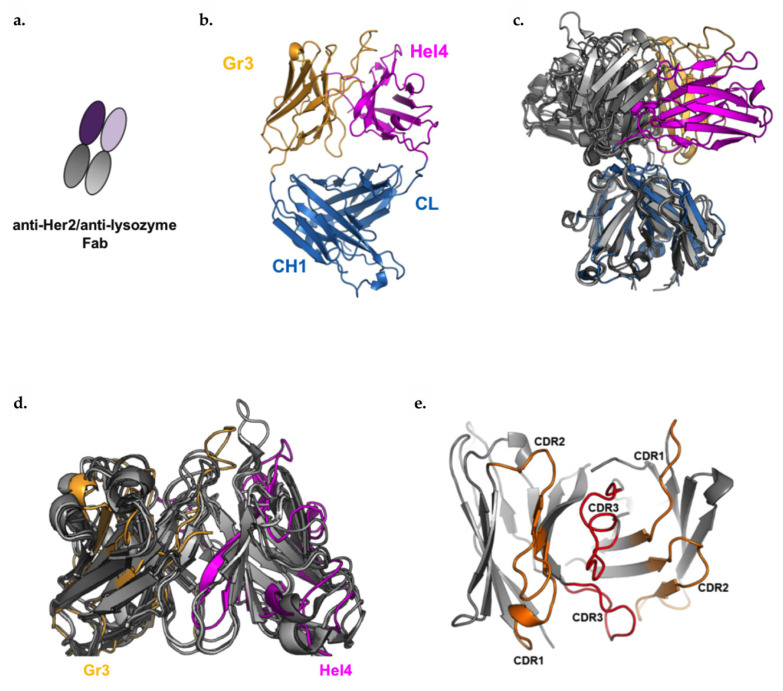
Crystal structure of 2xVH Fab shows VH:VH has a larger interface than natural VH:VL. (**a**) Schematic illustrating the configuration of VH domains in the 2xVH anti-Her2/anti-Lysozyme Fab. (**b**) A ribbon diagram illustrating the crystal structure of the 2xVH anti-Her2/anti-Lysozyme Fab, resolved to 2.55 Å. The Gr3 VH domain (shown in yellow) and the Hel4 domain (shown in magenta) were grafted to the CH1 and CL domains (shown in blue), respectively. (**c**) Superposition of the 2xVH (colored) with the following Fabs, derived from the VH 3-23 germline, in varying shades of gray: protein databank (PDB) 5I19, 5I1A, 5I1D, 5I1C. Structures are aligned on the CH1 domain. (**d**) Superposition of VH:VH domains from the 2xVH structure on canonical Fabs. (**e**) Top view of the VH:VH interface with CDR1 and 2 from both VH domains highlighted in orange and the long CDR3, thought to stabilize this interface, highlighted in red.

As mentioned previously, the heat maps in Figure 3a,b illustrate that the anti-lysozyme VH domain, Hel4, was one of the best tolerated VH domains in the 2xVH format, regardless of whether it occupied the VH_1_ or VH_2_ position within the molecule. To gain an understanding of why this VH domain was particularly amenable to the 2xVH format, we solved the crystal structure of the 2xVH anti-Her2/anti-lysozyme Fab schematized in Figure 6a, at a 2.55 Å resolution (Figure 6b).

In order to explore the differences between the 2xVH structure and a VH/VL pair on a standard IgG, this 2xVH Fab was compared to the canonical pairings of VH 3-23 with various VLs shown in Table 1 [51]. Superposition of the 2xVH Fab structure on this conventional VH3-23:VL Fab shows the VH:VH orient differently from the VH:VL domains with respect to CH1 and CL. When the structures are superimposed on the CH1 domain, the CH1:CL domains of both the canonical Fab and the 2xVH Fab overlay well until the elbow region of the IgG. Upstream of the elbow region, the VH:VH domains of the 2xVH Fab do not align with the direction of VH:VL domains as shown in Figure 6c. However, the VH:VH dimer alone (in absence of CH1/CL) shows good alignment with VH:VL *F_v_* domains (root mean square deviation, R.M.S.D, of structures tested = ~1–2 Å), indicating that while the orientations of the domains differ, the structure of the domains themselves do not (Figure 6d).

To understand why this 2xVH Fab construct may have favorable stability, we looked at the interaction interface between the two VH domains via the buried surface area, commonly used to estimate the interface between two domains or macromolecules. The buried surface area was calculated using PISA (Protein Interfaces, Surfaces and Assemblies) on CCP4 by averaging the contact surface of each VH domain [71]. VH:VL contact surface area values for VH3-23:VL structures were extracted from Teplyakov et al. [72]. The buried surface area of VH:VH in the 2xVH anti-lysozyme/anti-Her2 Fab is significantly larger than the typical VH:VL contact surface in the referenced Fab structures [51] (Table 1). The VH:VH interface in the 2xVH anti-lysozyme/anti-Her2 Fab structure is 1171 Å^2^, whereas the analogous VH:VL interfaces listed range from 684 to 828 Å^2^. Many of the interactions at the 2xVH anti-lysozyme/Her2 Fab interface are contributed by the long, 16aa CDRH3 of the anti-Her2 VH, Gr3, as shown in Figure 6e. This CDR3 shares a long interaction interface with CDRH2 in Hel4 VH while also interacting with additional residues from CDRH1. This large VH–VH interface area, though unique and highly dependent on the length of the CDRH3, appears to be characteristic of the format and likely plays a role in stabilizing the 2xVH anti-lysozyme/anti-Her2 Fab and contributing to the overall stability of the 2xVH format.

## 4. Discussion

IgG-like bispecific antibodies, as well as their multispecific counterparts, have become a highly desirable modality for clinical therapeutics [8,9]. An attractive promise of this format is its capacity for dual targeting—a feature allowing for the creation of molecules that generate novel function by simultaneously binding and/or eliciting interaction between their targets. While there are over one hundred different bispecific formats that attest to the importance of this dual targeting capability [10], the majority of IgG-like bispecifics require considerable engineering to eliminate mispaired or unwanted species [11]. Additionally, IgG-like bispecifics, unlike their monospecific counterparts, bind each of their two targets in a monovalent fashion and thus do not display avidity-induced enhancements in activity. Although bispecific formats like the two-in-one antibody design aim to create bivalency for each specificity [23], it has yet to be demonstrated whether concomitant interactions with both targets occur on a single Fab arm. Here, we have presented and characterized a new format, termed the 2xVH bispecific, that overcomes many of the engineering and manufacturing hurdles currently plaguing IgG-like bispecifics and offers a simple, unique and modular format capable of bivalent targeting. Additionally, this design holds significant promise for generating multispecific molecules in the future.

The 2xVH format utilizes two soluble, fully human autonomous VH domains, each with its own target specificity, to replace the native VH and VL pair within a traditional IgG. Since binding to each target is determined by a single domain, it eliminates the need for cognate chain pairing. Moreover, the molecules’ symmetry, with each half containing two specificities, gives rise to bivalency while retaining a nearly normal IgG structure. Consequently, these bispecific molecules can be produced via single-step purification methods and further polished for high purity via preparative SEC [11,37]. Previously, Shi and colleagues had developed a biparatopic 2xVH-like antibody targeting distinct and previously inaccessible epitopes on the β-Klotho/FGFR1c complex using two transgenic mouse-derived human sVH domains [37]. They showed that their biparatopic antibody was able to co-engage both epitopes of β-Klotho by biolayer interferometry (BLI) and inhibit downstream FGF21 signaling. However, BLI and other label-free optical analysis methods, which probe bulk changes in optical thickness at the biosensor tip, provide limited insight into whether dual binding events primarily occur on the same molecule at the same time and if so, whether these interactions are monovalent or bivalent in nature. Furthermore, the biparatopic anti-β-Klotho 2xVH molecules only make use of two VH domains, both derived from transgenic mice and targeting distinct epitopes on a single molecule [37]. Consequently, the capacity of this format to tolerate VH domains targeting two independent antigens was unexplored.

Given these limitations in the Shi et al. study, we searched the literature for publicly available VH domains that could be used to characterize and assess the versatility of the 2xVH format. After analyzing the expression, monomeric purity, thermal denaturation and binding properties of identified VH domains, we shortlisted seven different domains derived from both transgenic HcAb mice [32,34] and single domain VH phage display libraries [35,36]. Despite originating from different sources, all seven VH domains were derived from V3-23—a germline that is noted for its stability [49,51]. By grafting these seven VH domains into the 2xVH format in various configurations, we generated 31 unique 2xVH IgGs and 21 unique Fabs. While we observed that both phage display and transgenic mouse-derived VH domains could be substituted in the 2xVH format, the variability in expression and aSEC suggests that not every VH domain is well-tolerated. One of the best performing single VH domains, Gr3, showed greater than 90% monomeric purity by SEC, high *T_m_*s indictive of thermostability and a sub-nM binding affinity for its target, Her2 (Figure 2a). Nevertheless, when Gr3 was incorporated into the VH_1_ or VH_2_ position of the 2xVH Fab and IgG formats in combination with a number of other VH partners, many of these Her2-targeting molecules were 10 to 20% monomeric, suggesting a propensity for aggregation.

By contrast, Hel4, a thermostable VH domain targeting lysozyme [51], was able to rescue this phenotype, with the 2xVH anti-lysozyme/anti-Her2 IgG showing a four-fold improvement in expression and 93% monomeric purity (Figure 3). Previous work had shown that HEL4 contains a unique feature—a rotated framework tryptophan—that improved both the solubility and stability of HEL4 VH domain [51] and may be implicated in the favorable pairing of HEL4 with the less stable Gr3. More specifically, with its framework tryptophan no longer surface exposed, HEL4 may display a more attractive interaction interface for other VH domains including Gr3. This observation suggests that although VH domains grafted onto the IgG scaffold do not chain pair as do traditional VH and VL domains, paired VH domains do interact, as shown in Figure 6, and the VH–VH interface plays an important stabilizing role for the molecule. In the future, we hope to generate crystal structures for a larger subset of these 2xVH molecules and better understand both the stability of the VH:VH interface as well as possible steric hindrance in the presence of antigen binding. Nevertheless, our analysis of the anti-lysozyme/anti-Her2 2xVH molecule suggests that many of its characteristics, particularly its monomeric purity and aggregation propensity, are emergent to the 2xVH format rather than intrinsic to the constituent VH domains. This characteristic affords the possibility of designing VH libraries to strategically select VH domains pairs or engineering the VH–VH interface to accommodate a wide range of VH domains, including those, like Gr3, that show poor biophysical properties as single sVH.

In addition to characterizing the 2xVH constructs by aSEC, we also assessed individual molecules’ capacity to mediate binding with their target antigens as shown in Figure 4 and Figure 5. SPR analysis of 2xVH IgG shows that these molecules are capable of dual engagement. However, as previously mentioned, label-free optical analysis methods provide limited information on whether dual targeting can occur simultaneously on the same molecule or whether each binding interaction is spatially and temporally distinct. To truly assess if the 2xVH format can co-engage its two target antigens on a single Fab arm, we performed a label-based sandwich ELISA utilizing a 2xVH Fab targeting HA and human VEGF. Within this scheme, a fluorescence-based signal, recognizing a biochemical tag of interest, is only observed if the entire binding complex of the immobilized antigen, the 2xVH Fab and the second antigen bearing the tag can form. With our 2xVH anti-HA/anti-hVEGF Fab, we observed a dose-dependent fluorescence signal indicating that a single Fab molecule was able to bind both a surface-immobilized HA and a tagged human VEGF. This work characterizing simultaneous dual antigen binding by Fab molecules is the first definitive evidence that two antigens can be bound by a Fab arm on a single 2xVH molecule. This also opens the possibility for leveraging this modality to generate multispecific molecules [11] in the future. However, based on this report, it should be noted that the 2xVH format does not ensure coengagement of two targets by the two VH domains within each Fab arm in every case. Rather, each new pair of VH domains within the 2xVH format must be tested for coengagement of cognate antigens.

Beyond definitively demonstrating dual engagement, our binding studies with the anti-hVEGF VH domain, 5FV2, also validate the early characterization of bivalent and monovalent versions of this domain by Walker et al. [55]. In their work, Walker and colleagues describe a bivalent, dual dAb generated by linking two 5FV2 VH domains utilizing a Gly-Ser linker. Notably, this dual dAb was more effective at binding and capturing the VEGF homodimer than a single 5FV2 domain, a feature the authors attributed to the dual dAb engaging in a “side-on” or “pincer”-like capture of the VEGF homodimer and the generation of a 2:2 heteromeric complex [55]. In our analysis we see a similar synergistic effect associated with bivalency, where the VEGF partially dissociates from the monovalent, bispecific 2xVH anti-HA/anti-hVEGF Fab but not from the bivalent, bispecific 2xVH anti-HA/anti-hVEGF IgG regardless of the antigen binding order (Figure 4d,e). This synergism suggests that the geometry, and more particularly the mutlivalency, of VH domains in the 2xVH IgG can aid in the targeting of structurally complex antigens [37] and antigen homodimers [55].

The role of geometry and the synergistic binding arising from bivalency was an important feature not only for the 2xVH anti-HA/anti-hVEGF IgG but also in the anti-hTNFR1 and anti-VEGF 2xVH IgG molecules. While these two VH domains were tolerated in both the VH_1_ and VH_2_ positions of the IgG scaffold, their positioning influenced their capacity to sequentially engage target antigens (Figure 5). In fact, we saw ten-fold higher EC_50_ values, corresponding to a weaker dual target engagement, when the VH_1_ domain bound its target antigen first as opposed to when the VH_2_ domain initiated dual engagement. Though these observations are likely target dependent and were formulated based on a study of anti-hTNFR1 and anti-VEGF 2xVH IgG molecules, it has important implications for the design of clinically-relevant bispecific 2xVH where such sequential targeting may be required [12,73].

Our work highlights the potential versatility of a symmetric, simple yet novel bispecific format, the 2xVH. In addition to demonstrating that this format is amenable to two-chain expression with no engineering as well as single-step purification of both IgGs and Fabs, our work provides definitive evidence that a single 2xVH Fab can engage simultaneously with both its target antigens. This capacity for simultaneous dual engagement makes the 2xVH format a particularly attractive platform for the generation of bivalent, bispecific IgGs. Furthermore, when combined with technologies like “knob-into-hole” [25], orthogonal Fabs [55,74,75] or DuetMab [17], which increase the efficiency of heavy and light chain pairing, the bivalent, 2xVH technology affords the possibility of generating minimally engineered, asymmetric multispecifics including tri- or tetra-specific IgGs. In this vein, our work also illustrates the format’s enormous flexibility, keeping in mind caveats associated with domain position and VH–VH pairing, for incorporating VH discovered through both in vitro and in vivo discovery methods. Given the widespread use of both single domain phage libraries [36,40,59] and transgenic heavy chain mice [32,34,59], we anticipate that target-specific VH domains can be rapidly discovered. By leveraging tailored selections and immunizations, we envision the generation of panels of VH domains with diverse affinity and epitope targeting capabilities and the strategic incorporation of these domains within the 2xVH scaffold to create functionally enhanced, clinically relevant multispecific therapeutics.

## Figures and Tables

**Table 1:** Buried Surface Area in Fab Structures.

Construct	PDB	Contact Surface VH (Å^2^) or *VH1*	Contact Surface VL (Å^2^) or *VH2*	Interface (Å^2^)
H3-23:L1-39	5I19	795	817	806
H3-23:L3-11	5I1A	822	834	828
H3-23:L3-20	5I1C	670	698	684
H3-23:L4-1	5I1D	743	770	757
2xVH anti-Her2, Lysozyme	7JKB	1200	1143	1171

## Supplementary Materials

The following are available online at https://www.mdpi.com/2073-4468/9/4/62/s1, Figure S1: SPR-Based binding profiles for six VH domains, Figure S2: Exponentially Modified Gaussian (EMG) fits for four lead 2xVH molecules (Fabs and IgGs), Table S1: Crystallography Data for the 2xVH anti-lysozyme/anti-Her2 Fab.
