# Supplementary material for: Bringing the Heavy Chain to Light: Creating a Symmetric, Bivalent IgG-Like Bispecific
Source: Antibodies (Basel). 2020 Nov 6;9(4):62. doi: 10.3390/antib9040062 (PMC7709125; doi:10.3390/antib9040062)
Supplement: Supplementary file 1 [file antibodies-09-00062-s001.zip › Supplementary Figures_2xVH Paper.pdf]

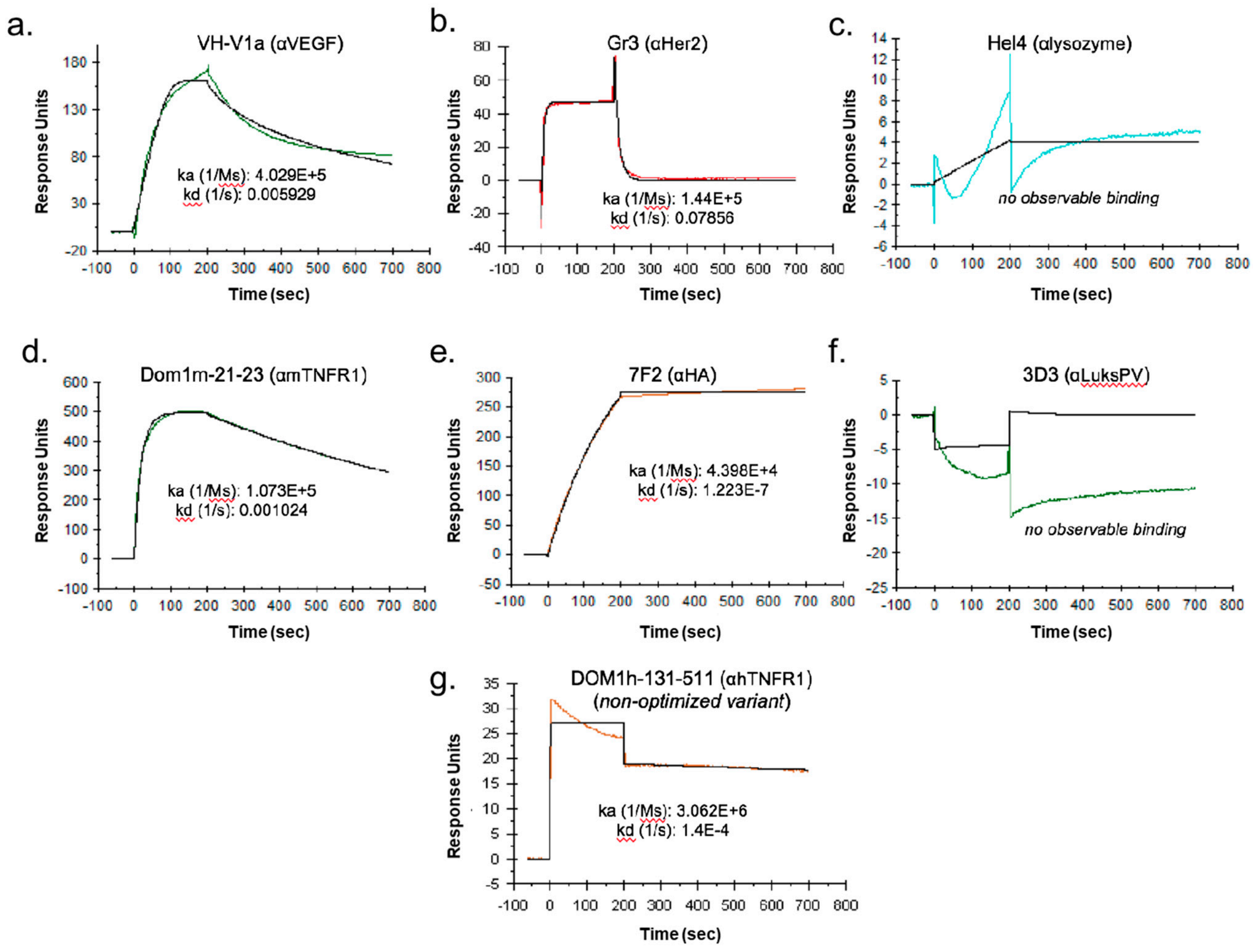


**Supplementary Figure 1. SPR-Based binding profiles for six VH domains**
Binding kinetics for expressible VH domains as assessed by SPR. VH domains were first captured on a chip using their C-terminal 6xhis-tag and their corresponding target antigen (**a-f**) was flowed over the chip at concentrations ranging from 0-50 nM. The target antigens included (**a**) VH-V1a (anti-VEGF) (**b**) Gr3 (anti-Her2) (**c**) Hel4 (anti-lysozyme) (**d**) Dom1m-21-23 (anti-mTNFR1) (**e**) 7F2 (anti-hemagglutinin) (**f**)3D3 (anti-LuksPV) (g) Dom1h-131-511 (anti-hTNFR1).

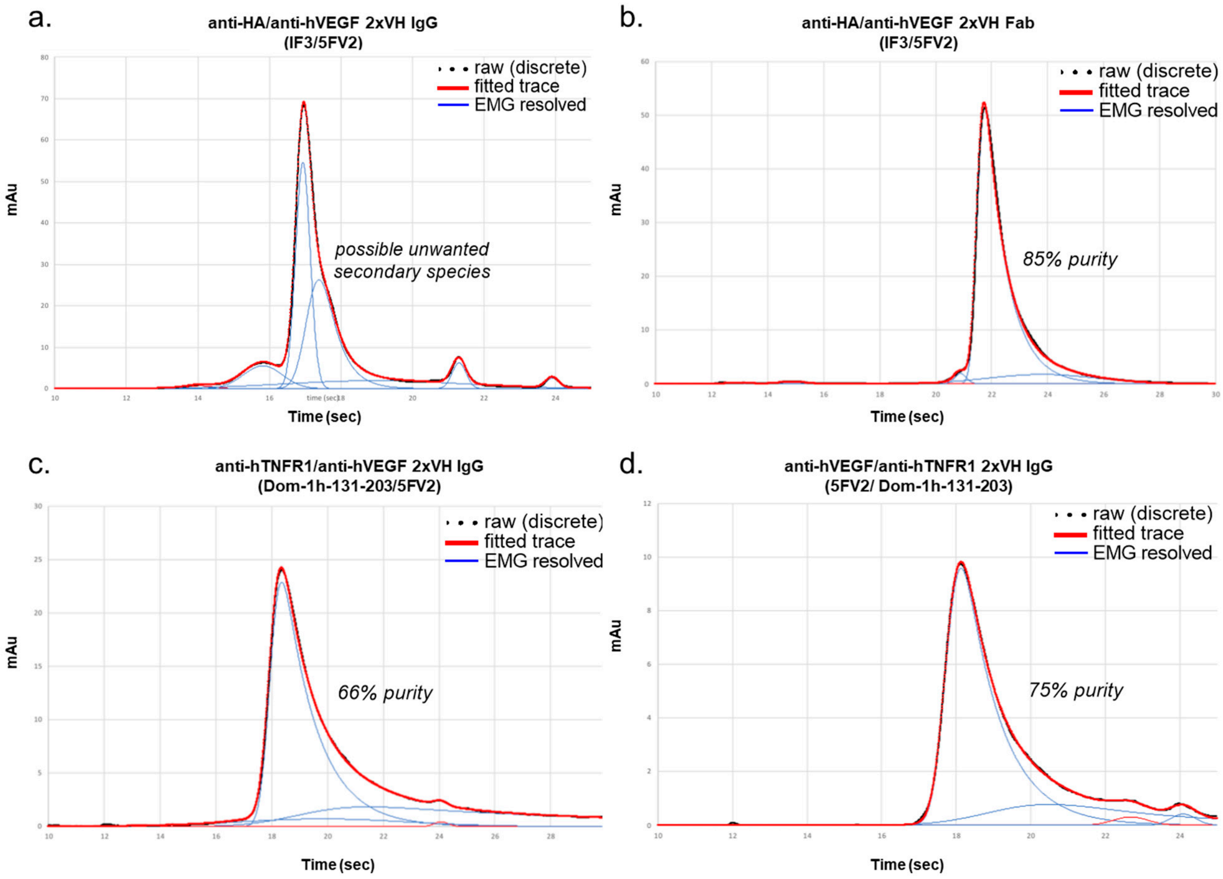


**Supplementary Figure 2. Exponentially Modified Gaussian (EMG) fits for four lead 2xVH molecules (Fabs and IgGs)**

Modified gaussian curves were fit to SEC traces for 2xVH molecules to more accurately determine the percentage of monomeric species and detect the presence of any low-molecular weight species. Traces and EMG fits are shown for (**a**) anti-HA/anti-hVEGF IgG (**b**) anti-HA/anti-VEGF Fab (**c**) anti-hTNFR1/anti-hVEGF 2xVH (**d**) anti-hVEGF/anti-hTNFR1 IgG.
